# Effect of cow's milk protein allergy during infancy on eating behavior at 4 years of age: A cohort study

**DOI:** 10.1002/jpn3.70348

**Published:** 2026-01-18

**Authors:** Anne Jardim‐Botelho, Marcela Barros Barbosa de Oliveira, Jackeline Motta‐Franco, Tatiane Graça Martins, Sarah Cristina Fontes Vieira, Ikaro Daniel de Carvalho Barreto, Aline Vieira Bezerra, Ricardo Queiroz Gurgel

**Affiliations:** ^1^ Federal University of Sergipe Aracaju Brazil; ^2^ Pediatrics of Federal University of Sergipe São Cristóvão Brazil; ^3^ Centro Brasileiro de Pesquisa em Avaliação e Seleção e de Promoção de Eventos (Brazilian Center for Research in Assessment, Selection, and Event Promotion – CEBRASPE Brasília Brazil; ^4^ Universidade Tiradentes (Tiradentes University) Aracaju Brazil

**Keywords:** children's eating behavior questionnaire, feeding difficulty, food fussiness, infant feeding

## Abstract

**Objectives:**

This study aimed to investigate the eating behaviors of preschool children who had been exposed to a restricted diet due to an oral food challenge‐confirmed diagnosis of cow's milk protein allergy (CMPA) during early infancy.

**Methods:**

This prospective cohort study compared the eating behaviors of Brazilian children previously diagnosed with CMPA to those of a nonallergic control group. Baseline data on infant feeding, clinical history, and sociodemographic characteristics were collected and later analyzed in association with the Children's eating behavior questionnaire (CEBQ) at 4 years of age. Linear regression models were used to assess associations between CMPA and CEBQ scores, with both crude and adjusted analyses performed.

**Results:**

A total of 74 children, with a mean age of 3.2 months at recruitment, were enrolled (30 with CMPA and 44 controls). Cesarean section delivery, rural geographic location, early introduction of substitute infant formula, and a family history of atopy were associated with higher food fussiness scores. After adjusting for early predictors of eating behaviors, the CMPA group scored significantly higher on the “desire to drink” scale (B adjusted: 2.61; *p* = 0.031) and on the “food fussiness” scale (B adjusted: 4.07; *p* = 0.017).

**Conclusions:**

Adherence to a CME diet during infancy, following an OFC‐confirmed CMPA diagnosis, was found to have a long‐term impact on eating behavior, as evidenced by higher scores on food fussiness and desire to drink scales, which are linked to feeding difficulties.

## Abstract (highlights)

**What is Known:**

- Feeding difficulties are commonly reported in children with food allergies.
- Cow's milk elimination diet can affect children's eating behaviors in developed countries.

**What is New:**

- Children from a resource‐constrained setting who had been exposed to a restricted diet due to an oral food challenge (OFC)‐confirmed diagnosis of cow's milk protein allergy (CMPA) during early childhood exhibited higher scores for Food Avoidance behavior at preschool age.
- Early‐life CMPA is associated with higher scores of food fussiness and desire to drink at 4 years of age, independent of the early predictors of these behaviors.

## Graphical abstract

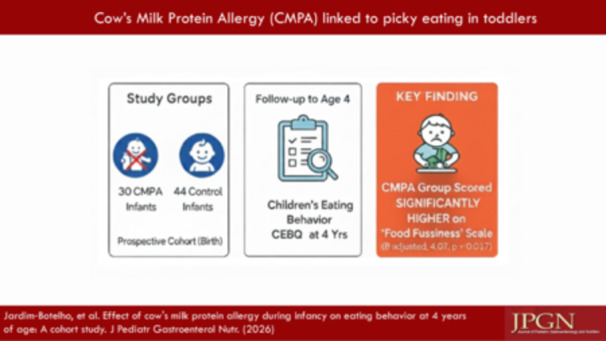

## 1 INTRODUCTION

Cow's milk protein allergy (CMPA), given that cow's milk is the primary nutritional source in the absence of breastfeeding, is the most prevalent food allergy in infants—affecting between 0.5% and 3% of young children in developed countries^1^ and approximately 1% of children aged 4–23 months in Brazil.^2^ Due to its ubiquity in Western diets, a cow's milk elimination (CME) diet can reduce dietary variety in infancy, thereby limiting sensory experiences. It is well established that the development of feeding skills occurs between 0 and 24 months of age, and limitations in flavor and texture exposure during this critical period may impair the development of oral‐motor functions essential for feeding, leading to inadequate eating behavior patterns throughout childhood.^3^, ^4^

Studies have shown that infants from high‐income countries who followed a CME diet exhibit higher scores of avoidant eating behavior during preschool and school‐age years^5^, ^6^ and, in contrast, a Thai study found that children aged 1–6 years with CMPA showed lower scores for SE and desire to drink, both behaviors associated with feeding difficulties.^7^ To date, however, no studies have longitudinally assessed the impact of early‐life CMPA on preschool eating behavior in children from Western developing countries. The aim of this prospective cohort study was to investigate the eating behaviors of preschool children who had been exposed to a restricted diet due to an oral food challenge (OFC)‐confirmed diagnosis of CMPA during early childhood (0–12 months). Our hypothesis is that children from resource‐constrained settings with an OFC‐diagnosed CMPA during infancy have significantly higher food selectivity scores than their healthy counterparts.

## 2 METHODS

### 2.1 Ethics statement

This study was approved by the Research Ethics Committee of the Federal University of Sergipe (CAAE 97662918.0.0000.5546) and conducted following the ethical principles outlined in the Declaration of Helsinki. Participation was voluntary, and caregivers were assured of the confidentiality of all information provided. Informed consent was obtained from all participants involved in the study.

### 2.2 Study design and participants

The data analyzed in this cohort study were collected as part of a longitudinal prospective investigation into the nutritional aspects of infants with CMPA in Northeastern Brazil. A detailed description of anthropometric measures and nutritional biomarkers is available in Jardim‐Botelho et al.^8^

The study included two groups: an experimental group composed of children with CMPA who had followed a CME diet for ≥6 months, and a control group consisting of children on an unrestricted diet and without symptoms suggestive of CMPA. Infants up to 6 months of age with symptoms of immunoglobulin E (IgE)‐mediated and/or non‐IgE‐mediated CMPA were prospectively recruited at Reference Center for Food Allergy of Sergipe (RCFAS) over a 12‐month period (February 2019 to February 2020). Healthy infants for the control group were recruited from a healthy child outpatient clinic. Recruitment took place between September 2019 and March 2020 (see Supplementary Material S1: Supplemental Digital Content 2). Inclusion criteria for both groups required children to be between 0 and 6 months of age at baseline. Specifically, the CMPA group required a diagnosis confirmed by OFC, while the control group had no allergy requirement.

Exclusion criteria for the CMPA group comprised prematurity, co‐allergy to other foods, and any other medical condition requiring a special diet. The control group was subject to similar exclusion criteria, specifically prematurity and any pre‐existing medical conditions.

At the conclusion of the follow‐up period, when the children were 4 years of age, we assessed parental reports for suspected or confirmed diagnoses of autism spectrum disorder (ASD); no positive cases were identified in either group.

Infants presenting with symptoms suggestive of CMPA (underwent an open OFC following symptom resolution after 4–8 weeks on a CME diet. This diet consisted of extensively hydrolyzed formula (eHF) or amino acid‐based formula (AAF), and/or maternal exclusion of all cow's milk protein (CMP) from the diet in breastfed infants.^9^, ^10^ Symptoms observed were predominantly gastrointestinal (diarrhea, blood in stool, reflux, vomit, and persistent constipation), along with behavioral and developmental signs (irritability, crying, refusal to feed, and faltering growth), and immediate allergic reactions (eczema, urticaria, angioedema, itchy eyes, coughing, and wheezing).

The OFC was conducted under medical supervision in accordance with the Practical Allergy Consensus (PRACTALL) guidelines.^11^ OFCs were not administered to patients with a clinical history suggestive of IgE‐mediated CMPA and serum‐specific IgE levels ≥5.17 kUA/L.^12^ Children who did not exhibit immediate reactions following OFC were instructed to regularly consume CMP. Families were advised to return to the allergy center at any time if delayed symptoms of CMPA occurred, in which case a clinical assessment would be performed. Children with delayed symptoms confirmed by a physician were also classified as OFC‐positive based on clinical criteria.

Infants in both groups received guidance from a pediatric dietitian regarding the appropriate introduction of complementary feeding at 6 months of age. Recommendations followed national and international guidelines to ensure dietary quality and diversity, introducing age‐appropriate foods and transitioning from purees to more textured foods by 10 months of age.^13^, ^14^

Nutritional deficiencies in children from both groups were assessed and managed individually at baseline and again at 2 years of age, based on the child's age and the specific nutrient deficiency identified.^15^, ^16^, ^17^, ^18^, ^19^ These data have been previously published in Jardim‐Botelho et al.^8^

At the beginning of the study, the children's parents were interviewed to obtain sociodemographic and clinical data using structured questionnaires. Data regarding household assets, parental income, access to water, and sanitation were used to construct a socioeconomic status index based on the methodology developed by the Associação Brasileira de Empresas de Pesquisa (ABEP), where “A” represents the highest socioeconomic level and “E” the lowest.^20^ Weight and height were measured following the standardized anthropometric protocol established by the World Health Organization (WHO), as detailed in Jardim‐Botelho et al.^8^

Four years after study enrollment, researchers contacted the children's caregivers by telephone to collect data on preschool‐age eating behavior using the validated Portuguese version of the children's eating behavior questionnaire (CEBQ).^21^, ^22^ The CEBQ consists of 35 items that assess eight subscales. Mothers were asked to rate their child's eating behavior using a five‐point Likert scale. The eight CEBQ subscales and their interpretations are as follows: Satiety responsiveness (SR), slowness in eating (SE), emotional under‐eating (EUE), and food fussiness (FF), which assess food avoidance behaviors; and food responsiveness (FR), enjoyment of food (EF), desire to drink (DD), and emotional overeating (EOE), which assess food approach behaviors. Higher scores reflect a stronger expression of either food approach or food avoidance tendencies.

At the time of the eating behavior assessment, all children in the CMPA group had developed spontaneous tolerance to CMP. Therefore, children in both the CMPA and control groups were consuming unrestricted diets and exhibited no symptoms suggestive of food allergy.

### 2.3 Data analysis

An a priori power analysis was performed using G*Power 3.1.9.7 for a two‐tailed Wilcoxon–Mann–Whitney test, assuming an effect size of 0.73, *α* = 0.05, and power = 0.80. The estimated sample size required was 74 participants, comprising 30 in the CMPA group and 44 in the control group.

All statistical analyses were conducted using R software (version 4.3.2),^23^ with a significance level of 5%. Linear regression models were used to assess associations between CMPA and eating behaviors, with both crude and adjusted analyses performed.^24^ Model assumptions were evaluated, including independence of residuals (Durbin–Watson test),^25^ homoscedasticity (Breusch–Pagan test),^26^ and normality of residuals (Shapiro–Wilk test).^27^ In cases where assumptions were violated, bootstrap techniques were employed to improve the robustness of the estimates.^28^ For each model, the regression coefficient (*B*), 95% confidence interval (CI), coefficient of determination (*R*² and adjusted *R*²), root mean square error (RMSE), and mean absolute error (MAE) were reported.^29^ To enable performance comparisons across variables with differing scales, RMSE and MAE were also expressed as a percentage of the maximum possible score for each predictor, following recent recommendations for standardized error reporting.^30^ This approach allowed identification of the dimensions with the most and least stable predictive capacity relative to their scoring range.

Statistical analyses were conducted using the rstatix, broom, olsrr, and the easystats suite. These packages collectively provided robust tools essential for assumption testing, regression diagnostics, and the calculation of effect sizes.^31^, ^32^, ^33^, ^34^ Detailed information regarding the implementation of these tools is available in the Supplementary Material S1 (see Supplemental Digital Content 2).

## 3 RESULTS

A total of 150 children evaluated at RCFAS met the inclusion criteria. Of these, 30 had OFC‐confirmed CMPA. An additional 44 healthy, age‐matched infants were recruited for the control group using a frequency‐matching protocol based on age.

CME diets were initiated in symptomatic infants at a mean age of 3.2 months ± 1.5 standard deviation (SD), and formal diagnoses were confirmed at a mean age of 5.4 months (SD 2.5). Gastrointestinal manifestations accounted for 90% of clinical CMPA presentations. Specifically, food protein‐induced allergic proctocolitis and food protein‐induced gastroesophageal reflux disease were diagnosed in 17 (56.7%) and 10 (33.3%) children, respectively. Acute urticaria/angioedema and anaphylaxis were observed in two (6.7%) children and one (3.3%) child, respectively.

The mean age of children at study entry was 2.8 ± 1.8 months, with no significant difference between the CMPA and control groups (*p* = 0.185). The mean age at the time of eating behavior assessment was 4.1 ± 0.2 years, again with no significant difference between groups (*p* = 0.596).

There were no significant differences in baseline anthropometric status between children with CMPA and those without allergies. However, when comparing *z*‐scores from baseline to the 18‐month follow‐up, children with CMPA showed significantly greater increases in weight‐for‐age and length‐for‐age compared with non‐allergic peers (regression coefficient [*δ*]: 1.511, 95% CI: 0.388–2.634; *p* = 0.008 and 2.120, 95% CI: 0.850–3.391, *p* = 0.001, respectively), as previously reported by Jardim‐Botelho et al.^8^

The demographic characteristics of the participants are presented in Table 1. Maternal age and educational level were significantly higher in the CMPA group (*p* = 0.033 and *p* = 0.005, respectively). Additionally, a greater proportion of children in the control group (77.3%) resided in urban areas compared with those in the CMPA group (53.3%) (*p* = 0.031).

**Table 1:** Demographic characteristics of the study participants.

	Cow's milk protein allergy group	Control group	*p* value
	*N* = 30	*N* = 44	
Age at enrollment (months)			0.186^a^
Median [IQR]	2.85 [2.27, 4.45]	2.73 [0.85, 4.43]	
Sex, *n* (%)			0.151^b^
Female	13/30 (43%)	27/44 (61%)	
Male	17/30 (57%)	17/44 (39%)	
Maternal age			0.033^a^
Median [IQR]	31 [24, 33]	24 [21, 33]	
Maternal education level, *n* (%)			0.005^b^
No formal education	3/30 (10%)	6/44 (13.6%)	
Primary school	2/30 (6.7%)	2/44 (4.5%)	
Secondary‐high school	12/30 (40%)	32/44 (72.4%)	
University	13/30 (43.3%)	4/44 (9.1%)	
Region of residence, *n* (%)			0.031^b^
Urban (capital)	16/30 (53.3%)	34/44 (77.3%)	
Rural (underserved area)	14/30 (46.7%)	10 (22.7%)	
Socioeconomic strata, *n* (%)			0.165^b^
A	1/30 (3.3%)	1/44 (2.3%)	
B1	4/30 (13%)	1/44 (2.3%)	
B2	8/30 (27%)	6/44 (14%)	
C1	3/30 (10%)	10/44 (23%)	
C2	5/30 (17%)	13/44 (30%)	
D‐E	9/30 (30%)	13/44 (30%)	
Number of siblings, *n* (%)			0.215^b^
None	14/30 (46.7)	27/44 (61.4%)	
1	8/30 (26.7%)	12/44 (27.3%)	
≥2	8/30 (26.7%)	5/44 (11.4%)	

Abbreviation: IQR, interquartile range.

^a^ Wilcoxon rank‐sum test.

^b^ Pearson's chi‐squared test with simulated *p*‐value (based on 2000 replicates).

^c^ Student's *t*‐test.

Details of participants' feeding and clinical characteristics at baseline are presented in Table 2. The prevalence of cesarean delivery was significantly higher among children with CMPA (80%) compared to the control group (45%) (*p* = 0.003). A family history of atopy was also more frequent among children with CMPA (76.7%) (*p* = 0.002). Rates of exclusive breastfeeding and breastfeeding up to 2 years of age were significantly higher in the control group (75% vs. 50%, *p* = 0.014; and 85.4% vs. 56.7%, *p* = 0.008, respectively).

**Table 2:** Baseline feeding and clinical characteristics of the study population.

	Cow's milk protein allergy group	Control group	*p* value
	*N* = 30	*N* = 44	
Birth weight (g)			0.800^c^
Median [IQR]	3295 [2850, 3480]	3200 [2865, 3450]	
Birth length (cm)			0.282^c^
Median [IQR]	49.0 [47.0, 50.8]	48.0 [46.5, 50.0]	
Mode of delivery, *n* (%)			0.003^d^
Cesarean	24/30 (80%)	20/44 (45%)	
Vaginal	6/30 (20%)	24/44 (55%)	
Infant feeding^a^			0.000^d^
Exclusive breastfeeding	14/30 (46.7%)	33/44 (75%)	
Substitute treatment formula^b^	16/30 (53.3%)	0/44 (0%)	
Standard formula	0/30 (0%)	11/44 (25%)	
Age at introduction of complementary food, *n* (%)			0.012^d^
No food offered	20/30 (67%)	41/44 (93%)	
5–29 days	3/30 (10%)	0/44 (0%)	
30–119 days	3/30 (10%)	2/44 (4.5%)	
120–179 days	4/30 (13%)	1/44 (2.3%)	
Number of foods offered by 9 months of age			0.658
Median [IQR]	11.5 [0, 16.0]	1.5 [0, 19.0]	
Breastfed up to age 2 years, *n* (%)			0.008^d^
Yes	17/30 (56.7)	35/41 (85.4)	
No	13/30 (43.3)	6/41 (14.6)	
Family history of atopy, *n*			0.002^d^
Yes	23/30 (76.7%)	18/44 (40.9%)	
No	7/30 (23.3%)	26/44 (59.1%)	
Weight‐for‐age *z*‐score			0.074^c^
Median [IQR]	−0.51 [−1.0, 0.4]	−0.03 [−0.52, 0.88]	
Length‐for‐age *z*‐score			0.113^e^
Median [SD]	−0.76 [0.84]	−0.50 [1.39]	
Weight‐for‐length *z*‐score			0.273^c^
Median [IQR]	0.46 [0.43, 0.86]	0.57 [0.18, 1.3]	

Abbreviations: IQR, interquartile range; SD, standard deviation.

^a^ Breastmilk/type of formula consumed at age <6 months.

^b^ Amino acid‐based or extensively hydrolyzed formula.

^c^ Wilcoxon rank‐sum test.

^d^ Pearson's Chi‐squared test with simulated *p*‐value (based on 2000 replicates).

^e^ Student's *t*‐test for independent samples.

At baseline, infants in the CMPA group were introduced to complementary foods earlier than those in the control group (33% vs. 7%, *p* = 0.012). Early complementary feeding was defined as the introduction of fruits or fruit juice, coconut water, flour‐based formula, cereals or tubers, meats/eggs, vegetables, biscuits, or commercially prepared baby food before the age of 6 months.

Demographic and baseline clinical characteristics, as shown in Tables 1 and 2, were associated with scores on the CEBQ (see Supplementary Material S2: Table S1). Children born via cesarean delivery and those living in rural areas showed significantly higher FF scores (14.0 vs. 11.5, *p* = 0.048 and 14.0 vs. 12.0, *p* = 0.011, respectively). Children who received substitute treatment formula for CMPA had lower FR scores (5.0 vs. 6.0 and 9.0, *p* = 0.004) and higher FF scores (16.5 vs. 11.0 and 12.0, *p* = 0.001) compared to their peers. Children with a family history of atopy had lower scores for FR and food enjoyment (6.0 vs. 9.0, *p* = 0.043; 8.0 vs. 11.0, *p* = 0.032, respectively), and higher scores for SR, EUE, and FF (9.0 vs. 8.0, *p* = 0.013; 7.0 vs. 5.0, *p* = 0.024; 14.0 vs. 12.0, *p* = 0.008, respectively). Children who were breastfed until the age of two had lower scores for SE, a behavior within the food avoidance domain (10.0 vs. 8.0, *p* = 0.007). Higher birth weight was associated with lower food enjoyment scores (*r* = −0.230, *p* = 0.045) and higher scores on the SE measure (*r* = 0.290, *p* = 0.013). Similarly, greater birth length was associated with higher SE scores (*r* = 0.250, *p* = 0.033).

The CEBQ scores at age 4 are presented in Table 3. The CMPA group scored significantly higher on SR, SE, EUE, FF and on the combined “avoidance eating behavior” construct (*p* < 0.005).

**Table 3:** Children's eating behavior questionnaire scores at age 4.

	Cow's milk protein allergy group *N* = 30	Control group *N* = 44	*p* value
	Median (minimum–maximum)	Median (minimum–maximum)	
Food approach	24.0 [19.0, 29.0]	24.0 [19.5, 28.0]	0.890
Food responsiveness	6.5 [5.0, 9.0]	8.0 [5.0, 10.0]	0.496^a^
Emotional over‐eating	2.0 [0.0, 4.50]	2.0 [0.0, 4.0]	0.835^a^
Food enjoyment	8.5 [6.0, 10.8]	10.0 [7.0, 11.0]	0.106^a^
Desire to drink	6.0 [3.0, 8.7]	4.0 [2.7, 6.3]	0.163^a^
Food avoidance	42.0 [35.0, 50.0]	31.0 [26.5, 35.5]	0.000^a^
Satiety responsiveness	9.5 [8.0, 11.8]	7.5 [6.0, 9.0]	0.004^a^
Slowness in eating	9.0 [8.0, 10.8]	7.0 [5.0, 9.0]	0.025^a^
Emotional under‐eating	8.0 [5.3, 9.8]	6.0 [4.0, 8.0]	0.051^a^
Food fussiness	16.0 [12.5, 19.5]	11.0 [8.0, 12.5]	<0.001^a^

*Note*: *N*, Valid cases.

^a^ Wilcoxon rank‐sum test.

Table 4 presents the results of both unadjusted and adjusted linear regression analyses examining associations between CMPA and the CEBQ scores, compared to children in the control group. In the unadjusted analysis, children with CMPA had higher scores for SR, SE, and FF by 2.0, 1.67, and 6.0 points, respectively, compared to the control group. The CMPA group also scored significantly higher on the combined food avoidance behavior construct than the control group (*B* = 11.4, *p* < 0.001).

**Table 4:** Association between children's eating behavior scores and CMPA: Unadjusted and adjusted linear regression analysis.

		Unadjusted		Adjusted^a^	
Dependent	Independent	*B* (95% CI)	*p* value	*B* (95% CI)	*p* value
Food approach		−1.45 (−6.24, 3.34)	0.549	3.57 (−4.11, 11.2)	0.356
Food responsiveness	CMPA/control	−0.77 (−2.62, 1.07)	0.406	1.64 (−1.22, 4.51)	0.255
Emotional over‐eating		−0.37 (−1.75, 0.88)^b^	0.566	0.20 (−2.10, 2.36)	0.845
Food enjoyment		−1.27 (−2.84, −0.29)	0.102	−0.92 (−3.48, 1.64)	0.473
Desire to drink		1.07 (−0.34, 2.44)^b^	0.116	2.61 (0.24, 4.97)	0.031
Food avoidance		11.4 (6.54, 16.2)	<0.001	5.19 (−2.02, 12.4)	0.155
Satiety responsiveness		2.02 (0.48, 3.45)^b^	0.014	0.64 (−1.85, 2.90)	0.610
Slowness in eating		1.67 (0.09, 3.25)	0.039	0.94 (−1.64, 3.51)	0.468
Emotional under‐eating		1.69 (−0.08, 3.47)	0.061	−0.35 (−3.11, 2.41)	0.800
Food fussiness		6.01 (4.00, 8.02)	<0.001	4.07 (0.74, 7.39)	0.017

Abbreviations: *B*, regression coefficient; CI, confidence interval (95%); CMPA, group of children with cow's milk protein allergy.

^a^ Linear regression adjusted for mode of delivery, region of residence, number of siblings, type of milk/prescribed formula, family history of atopy, breastfeeding until age two, birth weight, and birth length.

^b^ Models estimated via bootstrap due to assumption violations.

After adjusting the linear regression for variables associated with at least one eating behavior domain—mode of delivery, region of residence, number of siblings, infant feeding (breastmilk/type of formula consumed at age <6 months), family history of atopy, breastfeeding until age two, birth weight, and birth length—children with CMPA continued to show significantly higher FF scores than the control group (*B* = 4.07, *p* = 0.017). Also, they exhibited substantially higher DD scores (*B* = 2.61, *p* = 0.031).

To improve interpretability, RMSE and MAE values were rescaled as percentages of each variable's maximum possible range (see Supplementary Material S3: Table S2). The proportional error analysis revealed that the models with the best relative predictive performance were those using SR (RMSE = 12.5%, MAE = 10.5%) and FF (RMSE = 16.5%, MAE = 13.5%) as predictors. These subscales demonstrated lower prediction errors relative to their theoretical range, indicating stronger relative accuracy and model stability.

## 4 DISCUSSION

To our knowledge, this is the first longitudinal study to compare eating behavior, as measured by the CEBQ scales, between children with CMPA and healthy controls, while adjusting the analyses for a wide range of early‐life factors associated with CEBQ scores. Since participants were recruited from a birth cohort study, the effects of CMPA on current eating behavior could be assessed using prospectively collected data from early infancy.

The mean score for the combined food avoidance behavior construct was higher among children with CMPA, as were the subscales related to this behavior—with the exception of EUE—indicating a greater tendency toward feeding difficulties in children who experienced CMPA early in life. Similarly, Maslin et al. and Ercan et al. found higher scores for food avoidance behaviors in children with presumed or OFC‐confirmed CMPA following a CME diet.^6^, ^18^ In contrast to our findings, Ercan and Adiguzel and Kununya et al. reported lower SE scores in children with CMPA.^6^, ^7^ These discrepancies may be explained by the fact that those studies did not adjust their analyses for variables associated with children's eating behavior. In our study, the association between CMPA and higher SE scores was no longer significant after adjustment.

Unlike our results, Maslin et al. found no association between a CME diet and FF. This difference may stem from the cross‐sectional design of their study or from sociocultural differences, as the research was conducted in a developed country.^5^ Consistent with our findings, however, Ercan and Adiguzel observed that children with CMPA displayed more FF than their healthy peers.^6^

When the analyses were adjusted for variables associated with CEBQ subscales, FF remained significantly elevated in the CMPA group, and the DD subscale also became notably higher in these children. This is a noteworthy finding, as these two scales—FF and DD—may be related to feeding difficulties.^7^, ^35^ Selective eaters may attempt to meet their caloric needs through preferred drinks such as juice and milk. In line with this, Maslin et al. found that fussy eating and feeding difficulties were both associated with higher daily intake of milk substitutes. The authors suggested that this supports a straightforward dietary recommendation: reducing excessive formula consumption may stimulate appetite and improve mealtime behavior.^18^

After adjustment, the association between CMPA and the combined food avoidance construct was no longer statistically significant. However, the persistent association with FF and the emergence of a significant association with DD suggest that the condition itself may contribute to a predisposition for selective eating, independent of other cofactors linked to eating behavior.

The association between preschool FF and a history of CMPA may result from multiple factors, such as the elimination diet, which reduces dietary variety given that milk is a staple food in Western cultures^36^; the use of substitute treatment formulas, which may cause long‐term changes in taste preferences^37^; negative associations with food stemming from distressing feeding experiences related to allergic symptoms^38^, ^39^; and/or inappropriate feeding practices adopted by anxious parents.^3^, ^40^, ^41^ Another possible factor is impaired taste perception, which may contribute to food neophobia and be linked to compositional changes in the oral microbiota, as well as to the effects of chronic low‐grade inflammation from food allergies. This inflammation may disrupt the homeostasis of taste buds, reducing their number in both the circumvallate and fungiform papillae, and thus altering taste perception.^42^

This study has some limitations. First, parental feeding practices and styles, as well as specific details of complementary feeding, such as texture and variety, were not assessed, even though these factors can influence children's eating behavior. However, including family history of atopy in the adjusted model may have partially accounted for the bias introduced by parental feeding choices during the transition to solid foods. In addition, all participants received formal dietary counseling on complementary feeding practices following national and international guidelines.^13^, ^14^ Another limitation is that we did not evaluate maternal food preferences or the availability of different foods. Although this study reports differences in eating behavior, it does not include data on nutritional intake. Finally, while eating behavior was assessed through parental report rather than direct observation, the questionnaire used was validated, age‐appropriate, and demonstrated good internal consistency, reliability, and construct validity.^22^, ^43^

The strengths of this study include its prospective longitudinal design, the inclusion of a control group, and the homogeneity in age and sociocultural factors between the CMPA and control groups. Confirmation of CMPA through an OFC and a standardized elimination diet—excluding only cow's milk—in the CMPA group also represents a key strength. Additionally, adjusting the analyses for variables associated with the CEBQ subscales allowed us to isolate the effect of CMPA itself on children's eating behavior, independent of early dietary patterns and clinical or sociodemographic factors. The study met its recruitment target, and the proportional error analysis demonstrated strong relative predictive performance, particularly for the FF subscale.

Persistent fussiness can contribute to detrimental physical and psychological health outcomes, including nutritional deficiencies, faltering growth, and food‐related anxiety. Beyond these clinical risks, severe fussy feeding also causes considerable caregiver anxiety, frequently disrupts family mealtimes, and challenges overall family dynamics.^44^, ^45^, ^46^, ^47^ Fussy eating behaviors are not merely a transient “phase” but may follow a persistent trajectory. Results from a population‐based British cohort suggest that toddlers exhibiting higher levels of fussy feeding are also prone to experiencing subsequent increases in fussy feeding as they age.^48^ Therefore, our findings hold significant implications for researchers, clinicians, and the broader child and adolescent health community, given that the worldwide rise in food allergy prevalence may concurrently increase the risk of fussy feeding for which targeted interventions are needed.^49^, ^50^

## 5 CONCLUSIONS

Children from a resource‐constrained setting with CMPA exhibited higher scores for FF and a greater DD, independent of the variables associated with these behaviors. This suggests a potential negative impact of CMPA on eating behavior, even after the condition has resolved, and highlight the importance of timely and accurate diagnosis of CMPA to avoid unnecessary dietary restrictions or prolonged negative feeding experiences caused by allergic symptoms.

## CONFLICT OF INTEREST STATEMENT

The authors declare no conflicts of interest.

## Supporting information

Supplemental Digital Content 2.

Table S1.

Table S2.
