# Supplementary material for: Effect of cow's milk protein allergy during infancy on eating behavior at 4 years of age: A cohort study
Source: J Pediatr Gastroenterol Nutr. 2026 Jan 18;82(4):1119–28. doi: 10.1002/jpn3.70348 (PMC13050815; doi:10.1002/jpn3.70348)
Supplement: Supplementary file 1 — Supplemental Digital Content 2. [file JPN3-82-1119-s003.docx]

Supplemental Digital Content 2: **Expanded Methodological Details for Reproducibility**

This Supplemental Digital Content provides the specific methodological details of the R packages employed to ensure compliance with reproducibility standards for all data processing, statistical modeling, and reporting.

All analyses were conducted using R statistical software (version 4.3.2) (R Core Team, 2023). A robust workflow for data management, statistical modeling, and visualization was established using a comprehensive set of R packages. Core components, including dplyr, forcats, magrittr, ggplot2, and ggpubr from the tidyverse ecosystem, facilitated data cleaning, transformation, and initial exploratory visualization. Graphical outputs were refined using ggstatsplot, ggthemes, ggsci, hrbrthemes, and extrafont for precise aesthetic and typographical control.

rstatix, broom, olsrr, and the easystats suite were central to the statistical analyses, providing robust functionality for assumption testing, regression diagnostics, and effect size computation. For the integration of results into publication-ready formats, gtsummary, flextable, gt, and modelsummary were used to ensure the clear and consistent presentation of tables.

Additional packages, such as rio (for data import/export) and haven (for statistical software file handling), contributed to a unified and reproducible environment, thereby ensuring efficient analysis and high-quality output.

**References**:

Alboukadel, K. rstatix: Pipe-Friendly Framework for Basic Statistical Tests. 2023. R package version 0.7.2. [https://rpkgs.datanovia.com/rstatix/](https://rpkgs.datanovia.com/rstatix/). Accessed 29 Sep 2025.

Sjöberg, D. D. et al. Reproducible summary tables with the gtsummary package. The R Journal, v. 13, n. 1, p. 570-580, 2021. <https://doi.org/10.32614/RJ-2021-053>. Accessed 29 Sep 2025

Wickham, H. et al. Welcome to the tidyverse. Journal of Open Source Software, v. 4, n. 43, p. 1686, 2019. <https://doi.org/10.21105/joss.01686>. Accessed 29 Sep 2025.

Chamberlain, S.; WICKHAM, H. rio: A Swiss-army knife for data I/O. 2023. R package version 0.5.36. <https://cran.r-project.org/package=rio>. Accessed 29 Sep 2025.

Wickham, H. et al. dplyr: A Grammar of Data Manipulation. 2023. R package version 1.1.4. [https://CRAN.R-project.org/package=dplyr](https://cran.r-project.org/package=dplyr). Accessed 29 Sep 2025.

Bache, S. M.; WICKHAM, H. magrittr: A Forward-Pipe Operator for R. 2022. R package version 2.0.3. [https://CRAN.R-project.org/package=magrittr](https://cran.r-project.org/package=magrittr). Accessed 29 Sep 2025.

Wickham, H. ggplot2: Elegant Graphics for Data Analysis. 2. ed. New York: Springer, 2016.

Patil, I. Visualizations with statistical details: The ‘ggstatsplot’ approach. Journal of Open Source Software, v. 6, n. 61, p. 3167, 2021. <https://doi.org/10.21105/joss.03167>. Accessed 29 Sep 2025.

Kassambara, A. ggpubr: ‘ggplot2’ Based Publication Ready Plots. 2023. R package version 0.6.0. [https://CRAN.R-project.org/package=ggpubr](https://cran.r-project.org/package=ggpubr). Accessed 29 Sep 2025.

Wickham, H. forcats: Tools for Working with Categorical Variables (Factors). 2023. R package version 1.0.0. [https://CRAN.R-project.org/package=forcats](https://cran.r-project.org/package=forcats). Accessed 29 Sep 2025.

Makowski, D. et al. easystats: Framework for Easy Statistical Modeling, Visualization, and Reporting. 2023. R package version 0.6.0. <https://easystats.github.io/easystats/>. Accessed 29 Sep 2025.

Chang, W. extrafont: Tools for using fonts. 2022. R package version 0.19. [https://CRAN.R-project.org/package=extrafont](https://cran.r-project.org/package=extrafont). Accessed 29 Sep 2025.

Robinson, D.; HAYES, A. broom: Convert Statistical Analysis Objects into Tidy Tibbles. 2023. R package version 1.0.5. [https://CRAN.R-project.org/package=broom](https://cran.r-project.org/package=broom). Accessed 29 Sep 2025.

HEMELRIJK, J. et al. olsrr: Tools for Building OLS Regression Models. 2020. R package version 0.5.3. [https://CRAN.R-project.org/package=olsrr](https://cran.r-project.org/package=olsrr). Accessed 29 Sep 2025.

ARNOLD, J. B. ggthemes: Extra Themes, Scales and Geoms for ‘ggplot2’. 2021. R package version 4.2.4. [https://CRAN.R-project.org/package=ggthemes](https://cran.r-project.org/package=ggthemes). Accessed 29 Sep 2025.

XU, S.; WU, W. ggsci: Scientific Journal and Sci-Fi Themed Color Palettes for ‘ggplot2’. 2022. R package version 3.0.0. [https://CRAN.R-project.org/package=ggsci](https://cran.r-project.org/package=ggsci). Accessed 29 Sep 2025.

GOHEL, D. flextable: Functions for Tabular Reporting. 2023. R package version 0.9.3. [https://CRAN.R-project.org/package=flextable](https://cran.r-project.org/package=flextable). Accessed 29 Sep 2025.

ICHIUE, H. et al. gt: Easily Create Presentation-Ready Display Tables. 2023. R package version 0.9.0. [https://CRAN.R-project.org/package=gt](https://cran.r-project.org/package=gt). Accessed 29 Sep 2025.

AEILTS, B.; HESSE, C. modelsummary: Summary Tables and Plots for Statistical Models and Data. 2023. R package version 1.3.0. [https://modelsummary.com](https://modelsummary.com/). Accessed 29 Sep 2025.

RUDIS, B. hrbrthemes: Additional Themes, Theme Components and Utilities for ‘ggplot2’. 2020. R package version 0.8. <https://CRAN.Rproject.org/package=hrbrthemes>. Accessed 29 Sep 2025.

WICKHAM, H.; MILLER, E. haven: Import and Export ‘SPSS’, ‘Stata’ and ‘SAS’ Files. 2023. R package version 2.5.4. [https://CRAN.R-project.org/package=haven](https://cran.r-project.org/package=haven). Accessed 29 Sep 2025.
