# Supplementary material for: Effect of cow's milk protein allergy during infancy on eating behavior at 4 years of age: A cohort study
Source: J Pediatr Gastroenterol Nutr. 2026 Jan 18;82(4):1119–28. doi: 10.1002/jpn3.70348 (PMC13050815; doi:10.1002/jpn3.70348)
Supplement: Supplementary file 2 — Table S1. [file JPN3-82-1119-s001.docx]

**Table S1:** Factors associated with Children’s Eating Behavior Questionnaire scores in the study population.

|  | **FR** |  | **EOE** |  | **FE** |  | **DD** |  | **SR** |  | **SE** |  | **EUE** |  | **FF** |  |
| --- | --- | --- | --- | --- | --- | --- | --- | --- | --- | --- | --- | --- | --- | --- | --- | --- |
| **Characteristics** | **N = 74** | **p-value** | **N = 74** | **p-value** | **N = 74** | **p-value** | **N = 74** | **p-value** | **N = 74** | **p-value** | **N = 74** | **p-value** | **N = 74** | **p-value** | **N = 74** | **p-value** |
| **Mode of delivery** |  | 0.860 |  | 0.978 |  | 0.658 |  | 0.528 |  | 0.073 |  | 0.903 |  | 0.825 |  | **0.048** |
| Cesarean | 8.0 (5.0, 10.0) |  | 2.0 (0.0, 4.5) |  | 10.0 (7.0, 11.0) |  | 5.0 (3.0, 8.0) |  | 8.5 (7.0, 11.0) |  | 8.0 (6.0, 10.0) |  | 7.5 (3.0, 9.0) |  | 14.0 (11.0, 16.5) |  |
| Vaginal | 7.5 (5.0, 10.0) |  | 2.0 (0.0, 3.0) |  | 9.0 (7.0, 11.0) |  | 5.0 (3.0, 6.0) |  | 7.0 (6.0, 10.0) |  | 8.0 (5.0, 10.0) |  | 6.5 (5.0, 8.0) |  | 11.5 (9.0, 14.0) |  |
| **Region of residence** |  | 0.945 |  | 0.654 |  | 0.798 |  | 0.593 |  | 0.839 |  | 0.291 |  | 0.407 |  | **0.011** |
| Urban | 7.5 (5.0, 10.0) |  | 2.0 (0.0, 5.0) |  | 9.0 (7.0, 11.0) |  | 5.0 (3.0, 7.0) |  | 8.0 (6.0, 10.0) |  | 8.0 (6.0, 10.0) |  | 7.0 (4.0, 9.0) |  | 12.0 (8.0, 14.0) |  |
| Rural | 8.0 (5.0, 9.0) |  | 2.0 (0.0, 3.0) |  | 9.5 (7.0, 11.0) |  | 4.0 (2.5, 8.0) |  | 8.0 (6.0, 10.0) |  | 9.0 (7.0, 11.5) |  | 8.0 (5.0, 9.0) |  | 14.0 (12.0, 18.0) |  |
| **Number of siblings** |  | 0.417 |  | 0.320 |  | 0.614 |  | 0.695 |  | 0.273 |  | 0.203 |  | **0.011** |  | 0.088 |
| ≥ 2 | 9.0 (5.0, 10.0) |  | 3.0 (0.0, 5.0) |  | 9.0 (6.0, 10.0) |  | 5.0 (3.0, 7.0) |  | 9.0 (7.0, 11.0) |  | 9.0 (8.0, 11.0) |  | 9.0 (8.0, 12.0) |  | 15.0 (12.0, 21.0) |  |
| 1 | 6.0 (4.5, 9.0) |  | 1.5 (0.0, 2.5) |  | 8.0 (6.0, 11.0) |  | 5.0 (3.5, 8.0) |  | 8.5 (6.0, 12.0) |  | 8.5 (4.0, 10.0) |  | 5.0 (2.0, 8.5) |  | 12.0 (10.0, 14.0) |  |
| None | 8.0 (5.0, 10.0) |  | 2.0 (0.0, 4.0) |  | 10.0 (7.0, 11.0) |  | 4.0 (3.0, 7.0) |  | 8.0 (6.0, 10.0) |  | 7.0 (6.0, 10.0) |  | 7.0 (5.0, 8.0) |  | 12.0 (9.0, 15.0) |  |
| **Infant feeding** |  | **0.004** |  | 0.567 |  | 0.527 |  | 0.054 |  | 0.054 |  | 0.143 |  | 0.058 |  | **0.001** |
| Exclusive breastfeeding | 9.0 (5.0, 11.0) |  | 2.0 (0.0, 5.0) |  | 10.0 (7.0, 11.0) |  | 6.0 (4.0, 9.0) |  | 8.0 (6.0, 10.0) |  | 7.0 (5.0, 10.0) |  | 7.0 (4.0, 8.0) |  | 12.0 (9.0, 14.0) |  |
| CMPA treatment formula^1^ | 5.0 (4.0, 8.5) |  | 1.5 (0.0, 4.0) |  | 8.5 (4.5, 11.0) |  | 4.0 (2.5, 7.5) |  | 10.0 (8.0, 13.0) |  | 9.0 (8.0, 11.5) |  | 9.0 (5.5, 12.0) |  | 16.5 (13.0, 19.5) |  |
| Standard formula | 6.0 (2.0, 8.0) |  | 2.0 (0.0, 3.0) |  | 9.0 (6.0, 11.0) |  | 4.0 (2.0, 5.0) |  | 9.0 (4.0, 10.0) |  | 8.0 (6.0, 10.0) |  | 6.0 (0.0, 8.0) |  | 11.0 (8.0, 14.0) |  |
| **Family history of atopy** |  | **0.043** |  | 0.677 |  | **0.032** |  | 0.615 |  | **0.013** |  | 0.056 |  | **0.024** |  | **0.008** |
| Yes | 6.0 (4.0, 9.0) |  | 2.0 (0.0, 4.0) |  | 8.0 (6.0, 10.0) |  | 5.0 (2.0, 7.0) |  | 9.0 (7.0, 11.0) |  | 9.0 (7.0, 11.0) |  | 7.0 (6.0, 9.0) |  | 14.0 (10.0, 18.0) |  |
| No | 9.0 (5.0, 11.0) |  | 2.0 (0.0, 4.0) |  | 11.0 (8.0, 11.0) |  | 5.0 (4.0, 8.0) |  | 8.0 (6.0, 8.0) |  | 7.0 (5.0, 9.0) |  | 5.0 (3.0, 8.0) |  | 12.0 (10.0, 14.0) |  |
| **Breastfeeding until age 2** |  | 0.127 |  | 0.591 |  | 0.321 |  | 0.182 |  | 0.488 |  | **0.007** |  | 0.324 |  | 0.378 |
| Yes | 8.5 (5.0, 10.0) |  | 2.0 (0.0, 3.5) |  | 10.0 (7.0, 11.0) |  | 6.0 (3.0, 8.5) |  | 8.0 (6.0, 10.0) |  | 8.0 (5.0, 9.0) |  | 7.0 (4.0, 8.0) |  | 12.0 (9.5, 15.0) |  |
| No | 6.0 (5.0, 9.0) |  | 1.0 (0.0, 500) |  | 9.0 (7.0, 11.0) |  | 4.0 (3.0, 5.0) |  | 9.0 (6.0, 11.0) |  | 10.0 (8.0, 12.0) |  | 7.0 (5.0, 12.0) |  | 12.0 (10.0, 18.0) |  |
| **Birth weight (g)** | -0.150 | 0.191 | -0.022 | 0.851 | -0.230 | **0.045** | 0.057 | 0.632 | -0.130 | 0.286 | 0.290 | **0.013** | 0.086 | 0.467 | 0.098 | 0.407 |
| **Birth length (cm)** | -0.160 | 0.184 | -0.050 | 0.678 | -0.210 | 0.081 | 0.056 | 0.644 | -0.150 | 0.198 | 0.250 | **0.033** | 0.110 | 0.377 | 0.073 | 0.544 |

^1^Amino acid-based or extensively hydrolyzed formula.

**Legend:** FR – Food Responsiveness; EOE – Emotional Over-Eating; FE – Food Enjoyment; DD – Desire to Drink; SR – Satiety Responsiveness; SE – Slowness in Eating; EUE – Emotional Under-Eating; FF – Food Fussiness. CMPA: cow’s milk protein allergy.
