# Supplementary material for: Effect of cow's milk protein allergy during infancy on eating behavior at 4 years of age: A cohort study
Source: J Pediatr Gastroenterol Nutr. 2026 Jan 18;82(4):1119–28. doi: 10.1002/jpn3.70348 (PMC13050815; doi:10.1002/jpn3.70348)
Supplement: Supplementary file 3 — Table S2. [file JPN3-82-1119-s002.docx]

**Table S2:** Standardized effects and model fit for Children’s Eating Behavior Questionnaire (CEBQ) Dimensions in relation to cow’s milk protein allergy, adjusted for covariates.

| **Dependent Variable** | **B_std_**  **(CI 95%)** | **R²** | **Adjusted R²** | **RMSE** | **RMSE (%)** | **MAE** | **MAE (%)** |
| --- | --- | --- | --- | --- | --- | --- | --- |
| Food Approach | 0.35 (-0.41; 1.11) | 0.170 | 0.007 | 9.137 | 13.40 | 6.703 | 9.90 |
| Food responsiveness | 0.42 (-0.31; 1.16) | 0.224 | 0.071 | 3.407 | 17.00 | 2.640 | 13.20 |
| Emotional over-eating | 0.08 (-0.70; 0.86) | 0.124 | -0.049 | 2.874 | 24.00 | 2.171 | 18.10 |
| Food enjoyment | -0.28 (-1.06; 0.50) | 0.136 | -0.034 | 3.047 | 25.40 | 2.425 | 20.20 |
| Desire to drink | 0.83 (0.08; 1.59) | 0.179 | 0018 | 2.812 | 23.40 | 2.379 | 19.80 |
| Food Avoidance | 0.45 (-0.18; 1.09) | 0.427 | 0.314 | 8.584 | 12.60 | 6.905 | 10.20 |
| Satiety responsiveness | 0.18 (-0.52; 0.87) | 0.302 | 0.164 | 2.508 | 12.50 | 2.094 | 10.50 |
| Slowness in eating | 0.28 (-0.48; 1.04) | 0.166 | 0.002 | 3.064 | 25.50 | 2.308 | 19.20 |
| Emotional under-eating | -0.09 (-0.80; 0.62) | 0.272 | 0.129 | 3.286 | 20.50 | 2.486 | 15.50 |
| Food fussiness | 0.77 (0.14; 1.40) | 0.427 | 0.314 | 3.955 | 16.50 | 3.237 | 13.50 |

Legend: RMSE: root mean square error. MAE: mean absolute error. CI 95%: 95% Confidence Interval. B_std:_ Standardized Beta.
